# Globally important islands where eradicating invasive mammals will benefit highly threatened vertebrates

**DOI:** 10.1371/journal.pone.0212128

**Published:** 2019-03-27

**Authors:** Nick D. Holmes, Dena R. Spatz, Steffen Oppel, Bernie Tershy, Donald A. Croll, Brad Keitt, Piero Genovesi, Ian J. Burfield, David J. Will, Alexander L. Bond, Alex Wegmann, Alfonso Aguirre-Muñoz, André F. Raine, Charles R. Knapp, Chung-Hang Hung, David Wingate, Erin Hagen, Federico Méndez-Sánchez, Gerard Rocamora, Hsiao-Wei Yuan, Jakob Fric, James Millett, James Russell, Jill Liske-Clark, Eric Vidal, Hervé Jourdan, Karl Campbell, Keith Springer, Kirsty Swinnerton, Lolita Gibbons-Decherong, Olivier Langrand, M. de L. Brooke, Miguel McMinn, Nancy Bunbury, Nuno Oliveira, Paolo Sposimo, Pedro Geraldes, Pete McClelland, Peter Hodum, Peter G. Ryan, Rafael Borroto-Páez, Ray Pierce, Richard Griffiths, Robert N. Fisher, Ross Wanless, Stesha A. Pasachnik, Steve Cranwell, Thierry Micol, Stuart H. M. Butchart

**Affiliations:** 1 Island Conservation, Delaware Ave, Santa Cruz California, United States of America; 2 Ecology and Evolutionary Biology Department, University of California Santa Cruz, Santa Cruz, California, United States of America; 3 Royal Society for the Protection of Birds, The Lodge, Sandy, Bedfordshire, United Kigndom; 4 American Bird Conservancy, The Plains, Virginia, United States of America; 5 Institute for Environmental Protection and Research ISPRA and Chair IUCN Invasive Species Specialist Group, Via V. Brancati, Rome, Italy; 6 BirdLife International, Cambridge, United Kigndom; 7 Bird Group, Department of Life Sciences, The Natural History Museum, Tring, Hertfordshire, United Kigndom; 8 The Nature Conservancy, Nuuanu Ave, Honolulu, Hawai’i, United States of America; 9 Grupo de Ecología y Conservación de Islas, A.C. Av. Moctezuma, Zona Centro, Ensenada, B.C., Mexico; 10 Kaua`i Endangered Seabird Recovery Project, Hanapepe, Kaua`i, Hawai’i, United States of America; 11 John G. Shedd Aquarium, IUCN Iguana Specialist Group, S Lake Shore Dr, Chicago, Illinois, United States of America; 12 School of Forestry and Resource Conservation, National Taiwan University, Taipei, Taiwan; 13 Bermuda Zoological Society, Hamilton, Bermuda; 14 Island Biodiversity & Conservation center, University of Seychelles, Anse Royale, Mahé, Seychelles; 15 Nature Conservation Consultants Ltd, Gytheiou Chalandri, Greece; 16 Victoria, Mahé, Seychelles; 17 School of Biological Sciences, University of Auckland, Auckland, New Zealand; 18 Division of Fish & Wildlife, Commonwealth of the Northern Marianas, Lower Base, Saipan Commonwealth of the Northern Mariana Islands; 19 Institut Méditerranéen de Biodiversité et d'Ecologie marine et continentale, Aix Marseille Université, CNRS, IRD, Avignon Université, Centre IRD de Nouméa, Nouméa cedex, New-Caledonia; 20 Rinaldi Avenue, The Pines Beach, North Canterbury, New Zealand; 21 The Island Endemics Foundation, Boqueron, Puerto Rico, United States of America; 22 Palau Conservation Society, Palau Conservation Society, Bai Ra Maibrel, Koror, Palau; 23 Critical Ecosystem Partnership Fund, Crystal Drive, Arlington, Virginia, United States of America; 24 Department of Zoology, University of Cambridge, Cambridge, United Kigndom; 25 BIOGEOMED Group, University of the Balearic Islands, Cra, Valdemossa Balearic Islands, Spain; 26 Seychelles Islands Foundation, La Ciotat Building, Mont Fleuri, Victoria, Mahé, Seychelles; 27 Centre for Ecology and Conservation, University of Exeter, Cornwall Campus, Penryn, United Kigndom; 28 Sociedade Portuguesa para o Estudo das Aves, Avenida Columbano Bordalo Pinheiro, Lisboa, Portugal; 29 NEMO Srl, Piazza D'Azeglio, Florence, Italy; 30 Kennington-Roslyn Bush Road, Invercargill, New Zealand; 31 Oikonos Ecosystem Knowledge, Kailua, Hawai’i, United States of America; 32 FitzPatrick Institute of African Ornithology, University of Cape Town, Rondebosch, South Africa; 33 Sociedad Cubana de Zoología, La Habana, Cuba; 34 Stoney Creek Rd, Speewah, Queensland, Australia; 35 U.S. Geological Survey, Western Ecological Research Center, San Diego, California, United States of America; 36 BirdLife South Africa, Parklands, Johannesburg, South Africa; 37 Fort Worth Zoo, IUCN Iguana Specialist Group, Colonial Parkway, Fort Worth, Texas United States of America; 38 BirdLife Pacific, MacGregor Road, Suva, Fiji; 39 Ligue pour la Protection des Oiseaux, Fonderies Royales, 8 rue du Docteur Pujos, Rochefort, France; 40 Terres Australes et Antarctiques Françaises, rue Gabriel Dejean, Saint Pierre de la Réunion, France; University of Reunion Island, RÉUNION

## Abstract

Invasive alien species are a major threat to native insular species. Eradicating invasive mammals from islands is a feasible and proven approach to prevent biodiversity loss. We developed a conceptual framework to identify globally important islands for invasive mammal eradications to prevent imminent extinctions of highly threatened species using biogeographic and technical factors, plus a novel approach to consider socio-political feasibility. We applied this framework using a comprehensive dataset describing the distribution of 1,184 highly threatened native vertebrate species (i.e. those listed as Critically Endangered or Endangered on the IUCN Red List) and 184 non-native mammals on 1,279 islands worldwide. Based on extinction risk, irreplaceability, severity of impact from invasive species, and technical feasibility of eradication, we identified and ranked 292 of the most important islands where eradicating invasive mammals would benefit highly threatened vertebrates. When socio-political feasibility was considered, we identified 169 of these islands where eradication planning or operation could be initiated by 2020 or 2030 and would improve the survival prospects of 9.4% of the Earth’s most highly threatened terrestrial insular vertebrates (111 of 1,184 species). Of these, 107 islands were in 34 countries and territories and could have eradication projects initiated by 2020. Concentrating efforts to eradicate invasive mammals on these 107 islands would benefit 151 populations of 80 highly threatened vertebrates and make a major contribution towards achieving global conservation targets adopted by the world’s nations.

## Introduction

Global biodiversity loss is occurring at an unprecedented and undiminishing rate [1]. Invasive alien species have been a major driver of recent extinctions [2–4], and remain a serious threat to extant species [3, 5, 6]. Islands represent both a unique conservation need and opportunity. There are ~465,000 islands in the world [7], and though they comprise just 5.3% of the Earth’s terrestrial area, they have hosted 75% of known bird, mammal, amphibian and reptile extinctions since 1500 [4], and currently support 36% of species in these groups that are classified as Critically Endangered on the IUCN Red List [4]. Many of these animals are threatened as a direct consequence of invasive alien species, particularly non-native terrestrial mammals (hereafter “invasive mammals”) [3]. Invasive mammals, particularly cats (*Felis catus*) and rats (*Rattus* spp.), are the most damaging invasive species known on islands [2–5]. Eradication of invasive mammals from islands is a proven conservation tool [8], with clear evidence of subsequent native species recovery [9, 10]. More than 1,200 invasive mammal eradications have been attempted on islands worldwide, with an average success rate of 85%. In addition, larger, more remote and technically challenging islands are being successfully cleared of invasive species populations each year [8, 11].

Invasive mammal eradications on islands are significantly contributing towards achieving global conservation commitments agreed under the Convention on Biological Diversity [12] (2020 Aichi Targets 9—invasive alien species control or eradication, and 12—preventing threatened species extinctions) and United Nations Sustainable Development Goals [13] (targets 15.5—halt the loss of biodiversity, and 15.8—prevent and reduce the impact of invasive species on land and water ecosystems). Formal funding mandates by the Global Environment Facility [14], and commitments by nations striving to meet global targets (e.g. [15–17]) further support invasive mammal eradications on islands. However, the accelerated pace of biodiversity loss, and the global scale of need for invasive mammal eradications on islands, requires considerable financial resources, political will and stakeholder support [18, 19], thereby necessitating identification of the most important and achievable projects. A key obstacle to identifying global eradication priorities has been the availability and resolution of threatened native and invasive insular species distribution information, along with explicit criteria on eradication feasibility, including both technical criteria and socio-political acceptability. Socio-political feasibility, defined here as the combined social and political factors influencing acceptability of conservation actions, is critical for invasive mammal management and has not been previously incorporated into a global evaluation framework for eradications [18, 20]. Prior to this study, the only systematic global assessment of islands for invasive mammal eradication was restricted to only 367 islands for 130 globally threatened bird species, used incomplete breeding distribution data and did not consider socio-political factors or eradication timeframe [21].

We combined conservation opportunity and feasibility into a unified framework for identifying globally important islands where eradication of invasive mammals can benefit globally threatened birds, reptiles, mammals and amphibians. To achieve this, we built upon a recent global database on island characteristics and the distribution of threatened vertebrates and invasive species [22, 23] allowing comprehensive analyses to identify islands where vertebrate extinctions can be prevented by eradicating invasive mammals. We then 1) determined globally important islands where the eradication of invasive mammals can benefit native amphibian, bird, reptile and mammal species listed as Critically Endangered or Endangered on the IUCN Red List (hereafter, “highly threatened vertebrates”), 2) identified which islands meet current technical feasibility criteria, and 3) evaluated if eradication planning or operations are socio-politically feasible to initiate by 2020, 2030 or not in the foreseeable future. Our results provide a global assessment of important conservation opportunities and support regional and national decisions about where and how to prevent extinctions.

## Methods

We identified globally important islands for invasive mammal eradication to benefit highly threatened vertebrates using a systematic framework (Fig A in S1 File). We first determined the relative conservation value of all islands that support highly threatened vertebrates and are negatively impacted by invasive mammals present. Second, we determined island importance for invasive mammal eradication by considering the: a) conservation risk reduction from conducting eradications on technically feasible islands (hereafter eradication benefit [EB] [21, 24]) using the same general procedure as [24]; and b) socio-political feasibility of conducting an eradication on these islands. Islands with the largest EB were considered of highest importance for eradication. Socio-political feasibility was evaluated for each island where EB > 0, and we identified when it would be feasible to initiate an invasive mammal eradication project: by 2020, 2030, or not in the foreseeable future, based on review by experts with local and regional knowledge (see below for further details). The final list represents those islands where eradication projects could be feasibly initiated by 2020 or 2030. We considered the initiation of an eradication to include planning phases, and not just operational implementation, because these preliminary activities can take considerable time to achieve and represent a demonstrable commitment towards completing the eradication.

### General data collection

Data describing the distribution of highly threatened vertebrates breeding on islands, invasive mammals on these islands, and impacts of invasive mammals to highly threatened vertebrates were collated from the International Union for the Conservation of Nature (IUCN) Red List, BirdLife International’s species factsheets (www.birdlife.org), the Global Invasive Species Database (www.iucngisd.org), and the Threatened Island Biodiversity database [22, 23]. We summarize the data collected and, include definitions of the parameters used. We included highly threatened vertebrates based on conservation status on the IUCN Red List (2013.1). Marine mammals and sea turtles were excluded. We included species with breeding distributions classified as either insular or continental and insular, and where breeding status was defined as confirmed, probable or potential (based on definitions in [23]). Note that we purposefully masked islands that were considered sensitive locations as identified by experts, or if they were <100 ha and contained a reptile species, in order to limit the distribution of information that could be used for illegal wildlife trafficking. The island breeding distributions of highly threatened vertebrates and invasive mammals are tabulated in the Threatened Island Biodiversity database (available online at http://tib.islandconservation.org).

We defined invasive mammals as terrestrial vertebrate species whose introduction or spread by direct human action outside their natural distribution has been documented as negatively impacting native biodiversity [25]. To identify invasive mammals for our study, we first identified non-native mammals co-occurring with highly threatened vertebrates on the same island. We considered each non-native mammal and highly threatened vertebrate species co-occurrence as an interaction and classified the potential negative impact of that interaction as confirmed, suspected or none (see definitions in Table A in S1 File). For highly threatened birds, impact was based on the timing, scope and severity of invasive species threats [26]. For highly threatened reptiles, amphibians and mammals, impacts were derived from the literature, an expert review and additional data from a UK Overseas Territories assessment [24]. For invasive mammal presence on islands, we included records in our analyses if presence was confirmed or suspected (based on definitions in [23]), including if they were subject to ongoing eradication operations because the removal of these populations had not yet been assured at the time of analysis. Where known, for each island we identified if the invasive mammal population was feral and sought to exclude populations from analyses if they were entirely domestic or farmed (e.g. cattle, goats). Where status was unclear or information was missing we took a conservative approach and assumed these populations were feral and a threatening process was present. We limited our analysis to invasive mammals because it is well demonstrated that they have significant negative impacts on a broad range of vertebrate species [2–6], and there are well established examples and techniques for their successful eradication [8, 11]. Although other invasive alien species (e.g. plants, birds, reptiles, and insects) can also negatively affect biodiversity and threatened species with extinction, these were excluded from our analyses because there are fewer examples of successful eradication (e.g. see [27]).

We expect the list of important islands generated from our study to be an underestimate due to knowledge gaps describing the distribution for some globally threatened species and invasive mammals on islands. Similarly, updates to the IUCN Red List may include revisions to species’ extinction risk category and taxonomy, the inclusion of which may change the results of this analysis (see [23] for full review). Future efforts to identify important islands for invasive mammal eradication can re-apply these described methods to generate an updated list of islands.

### Identifying important islands for invasive mammal eradication

#### Step 1a: Current Conservation Risk (CCR)

To determine conservation priority, we calculated Current Conservation Risk (CCR), adapted from [21] and [24] and defined as the degree of conservation risk from invasive mammals impacting highly threatened vertebrates on an island. This term is synonymous with the term “potential conservation value” applied in [21] and [24]. We took an inclusive approach and considered those islands with potential breeding populations of highly threatened vertebrates as equal to those with confirmed or probable breeding populations. For each highly threatened vertebrate population, we calculated the product of extinction risk, irreplaceability and severity of impact of the most harmful invasive species on a given island and summed these to determine CCR for each island. Overall, the CCR was calculated as

$${CCR}_{i}=(\sum_{1}^{s}E_{s,i}\times I_{s,i}\times Z_{s,i})$$ (Eq 1)

where *E* is the extinction risk of each native species *s* occurring on island *i*; *I* is the irreplaceability of each native species’ global importance on a particular island, and *Z* is the maximum severity of impact of any alien species on island *i* on the native species *s*.

*Extinction Risk* (E) followed [21], and was set as 0.5 and 0.05 for CR and EN species, respectively. This method followed [28] on the basis of the relative extinction risk associated with the respective IUCN Red List categories, based on the quantitative thresholds for each of the Red List criteria. Changing this classification to a linear scoring system following [24] (i.e. scoring 4 for CR and 3 for EN) did not substantially affect the ranking of islands, with most of the top 20 islands being identical under both approaches. A more detailed quantitative estimate of extinction risk, as required by other prioritization approaches (e.g. [29, 30]), was not available for most species considered here.

*Irreplaceability* (I) was calculated as 1/total number of all islands a species breeds on, regardless of island area [21]. For species with both insular and continental populations (n = 93), the estimate for *I* does not take account of the continental population. This is justifiable because islands may offer the most viable opportunity to provide invasive mammal-free habitat for these species (S1 File).

*Severity of negative impact* (Z) estimated the maximum negative impact each invasive mammal had on each highly threatened vertebrate species on a given island, and was based upon a previously published framework [24]. Negative impacts were scored as 2 (confirmed impact), 1 (suspected impact), or 0 (no evidence of impact, or confirmed no impact). If no impact data were available for a species, we assumed that maximum impacts recorded for a highly threatened vertebrate by an invasive mammal would be analogous to other highly threatened vertebrates in the same taxonomic family. Despite this, we acknowledge that there may be impacts not accounted for in our study, because the effects of some invasive mammals, such as invasive mice (*Mus* spp.) [31], are less understood. To assess the potential impact of rodents being present on islands where status is unknown, we re-ran analyses assuming they were present (S1 File).

#### Step 1b: Potential Conservation Risk (PCR)

Having determined the greatest potential conservation gains from eradication (CCR), we reduced this list of islands to include only those where eradication was technically feasible (Potential Conservation Risk or PCR).

To inform PCR, we first assessed eradication feasibility for each invasive mammal species on each island, using thresholds for human population size and island area for each invasive mammal type (S1 File and Table B in S1 File). These two factors significantly limit the feasibility for invasive mammal eradications and are useful for large geographic scale comparisons, such as in this study [32, 33]. Area and human population thresholds were extracted from the Database of Islands and Invasive Species Eradications (accessed in March 2015 and October 2016) [11] for invasive mammals with demonstrated precedent for successful eradication, and for eradications currently in progress or planned, and reviewed by expert practitioners (S1 File).

If an invasive mammal was deemed feasible to eradicate from an island, the severity of negative impact score (*Z*) was set to zero because that negative interaction would no longer occur on the island once the invasive mammal was eradicated. If an invasive mammal was not deemed feasible to remove, the threat score remained the same. We then calculated PCR for all islands using the same equation as for CCR (Eq 1). CCR therefore differed from PCR only for those islands where the feasible removal of invasive mammals led to a lower maximum severity of impact (*Z*) for at least one highly threatened vertebrate. Finally, we calculated an eradication benefit (*EB*) score for each island by subtracting PCR from CCR. Higher EB values equaled greater risk reductions and this value was applied as the primary ranking of islands. Where more than one invasive mammal presented impacts on the island, the complications of eradicating only a subset of invasive mammals were not assessed, but understanding these interactions would be necessary for finer-scale feasibility assessments [29].

Including cost can improve the utility of conservation prioritizations [34]. In general, the operational cost of invasive mammal eradication is expected to increase with island size, the number of invasive species targeted, and whether the island has permanent human settlements. However, total project cost can vary widely across different socio-political geographies due to other factors (e.g. regulatory compliance, which methods are legally sanctioned, local cost of supplies), and may introduce variance greater than operational costs alone [35]. Hence, including operational cost estimates for all islands in our analyses would misrepresent total project cost and produce inaccurate outcomes. However, to support our results, for projects identified as feasible to initiate by 2020, we highlight those expected to be the least expensive to implement, based on island size (<100 ha), absence of permanent human habitation, and islands where eradicating only one or two invasive mammal species would benefit highly threatened vertebrates.

#### Step 2: Socio-political feasibility

For those islands deemed technically feasible in Step 1, we conducted a systematic assessment of the socio-political feasibility of invasive mammal eradication. As data are not available to quantify socio-political feasibility world-wide, we chose to use an expert-elicitation method to assess feasibility based upon practitioners’ knowledge. Between 2015–2017, we sent data to 116 conservation practitioners with expertise within the region of interest. Each expert was asked to assess feasibility to initiate an invasive mammal eradication project within their area of expertise by 2020, 2030 or not in the foreseeable future. We considered the eradication of all invasive mammal populations concurrently within the same timeframe. Each expert received a questionnaire, which addressed: 1) the history of previous invasive vertebrate eradications on islands and capacity for invasive vertebrate eradications in the country or territory, 2) political acceptability at a national or territory level, 3) influence of permanent human habitation on any proposed invasive mammal eradication, and 4) social acceptability at the local scale (S1 File).

We prompted experts to consider these four factors so that their answers regarding timeline were evaluated in a consistent manner. The first two considerations operate on a national / territorial scale, and the last two factors operate on a local scale, and in some cases local-scale considerations could override national scale factors. If there was disagreement among experts on the potential timeframe to initiate an eradication, we sought to obtain consensus through subsequent communication, or used the most conservative (i.e. later) timeframe when consensus was not achievable. Some experts also considered technical feasibility, including re-invasion risk and island size, in their consideration of timeframe. We expect that timescales identified to initiate eradications may change with changes in socio-political circumstances, and thus feasibility assessments would then need to be re-evaluated. Those identified as socio-politically feasible to initiate by 2020 or 2030 may not be exactly initiated by these timelines if resources and political will prove lacking, but we do expect that, on average, eradications identified in the 2020 timeframe will be more feasible sooner rather than those identified in the 2030 timeframe.

To provide context for the socio-political assessment and provide a metric for the expertise applied to the analysis, we collected data on the experts contributing to this assessment. We asked experts to tell us how many years they had spent actively working (e.g. research, planning, implementing) in conservation 1) in any capacity; and 2) dealing with the threat of invasive alien species.

## Results

### Highly threatened vertebrates and invasive mammals on islands

We examined 1,279 islands with 2,823 populations of 1,184 species of highly threatened vertebrates (318 amphibians, 282 reptiles, 296 birds and 288 mammals). Islands (median size = 430 ha) ranged in size from <0.1 ha (numerous islands and rockstacks) to 783,400 km^2^ (New Guinea). Most islands supported only one population of a highly threatened vertebrate (median 1, range 1–155 populations per island), and most of these populations were single-island endemic species (median 1, range 1–77 islands per population). Most islands (58%, n = 743) also had one or more of 184 species of non-native mammals (median = 4, range 1–34).

On these 743 islands with invasive mammals, we identified 17,313 potential interactions among the 184 species of non-native mammals co-occurring with highly threatened vertebrate populations. Of the 184 non-native mammal species, 47 (26%) had at least one interaction that was suspected or confirmed to negatively impact a highly threatened vertebrate on 574 islands. These non-native mammal species were thus considered equivalent to invasive mammals for the remainder of our study. In total, 3,990 negative interactions were identified, with 83% involving just eight invasive mammal taxa: feral cats and dogs, rats (three species), small indian mongoose (*Herpestes auropunctatus*), pigs (*Sus scrofa*) and goats (*Capra hircus*) (Fig B in S1 File). We found 260 islands (20% of the 1,279 islands with highly threatened vertebrates) that appeared to be free of invasive mammals, with 94 (36%) of these islands having successful eradications completed previously. Protecting these islands requires effective biosecurity policies and procedures to prevent invasive species–particularly the most damaging taxa (Fig B in S1 File)–from becoming (re)established [36].

### Important islands for invasive mammal eradications

Of the 574 islands where highly threatened vertebrates and invasive mammals co-occur, we identified 292 islands that met feasibility criteria of human population size and island area, and where an impact from an invasive mammal on a highly threatened vertebrate could potentially be removed by eradication.

For 218 of 292 islands (75%), we received input from 54 experts (47% response rate) regarding socio-politically feasibility to initiate an eradication by 2020, by 2030, or not in the foreseeable future (S1 Data File). These experts had between 5 and 49 years (median = 22) of expertise in conservation and a cumulative total of >804 years experience dealing with the threat of invasive alien species. We received no response, or experts classified socio-political feasibility as unknown, for 74 islands, which were removed from subsequent results but are identified as a priority data gap (S2 Data File).

**Fig 1:**
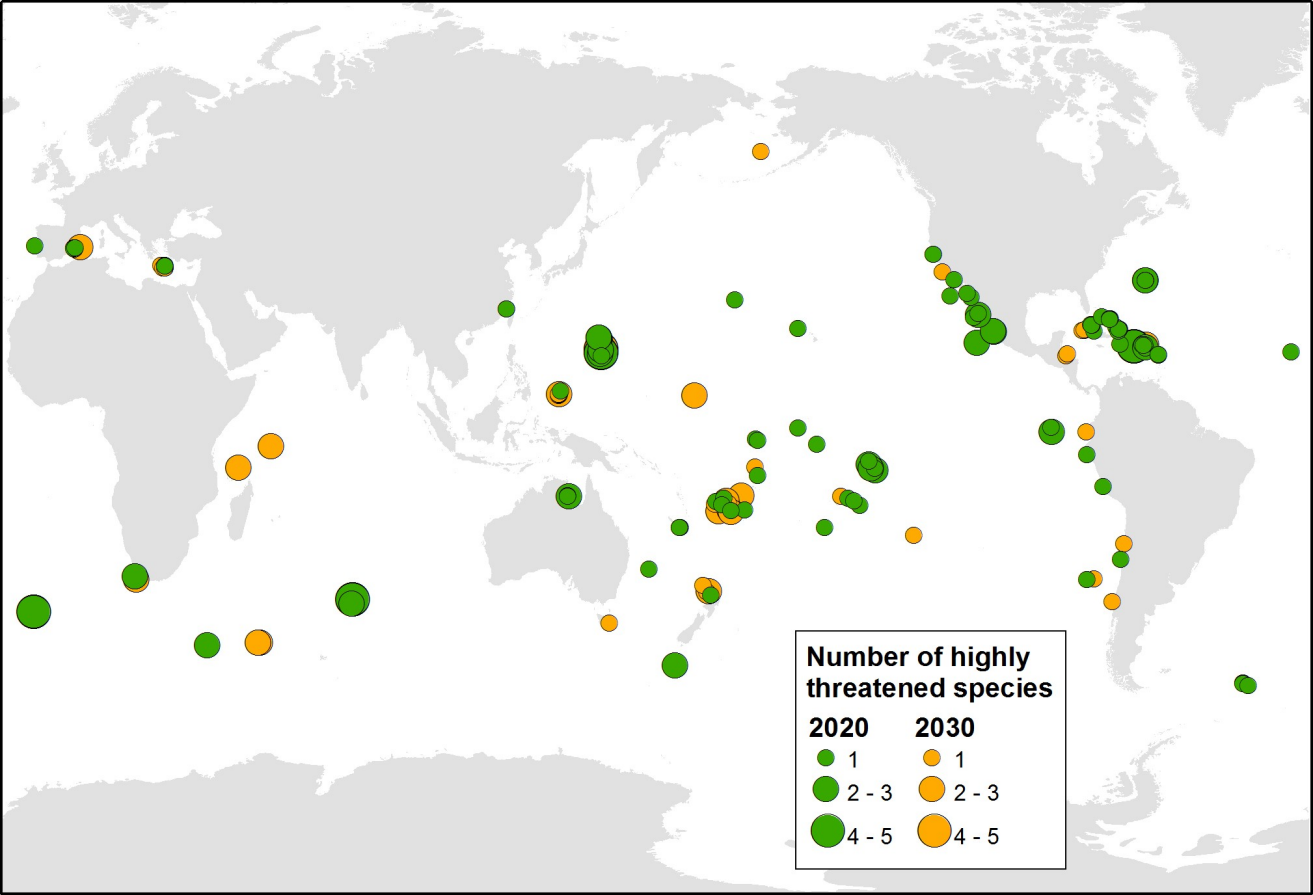
The location of the 169 highest-ranked islands where eradication of invasive mammals could feasibly be initiated by 2020 or 2030 to benefit highly threatened vertebrates.

**Fig 2:**
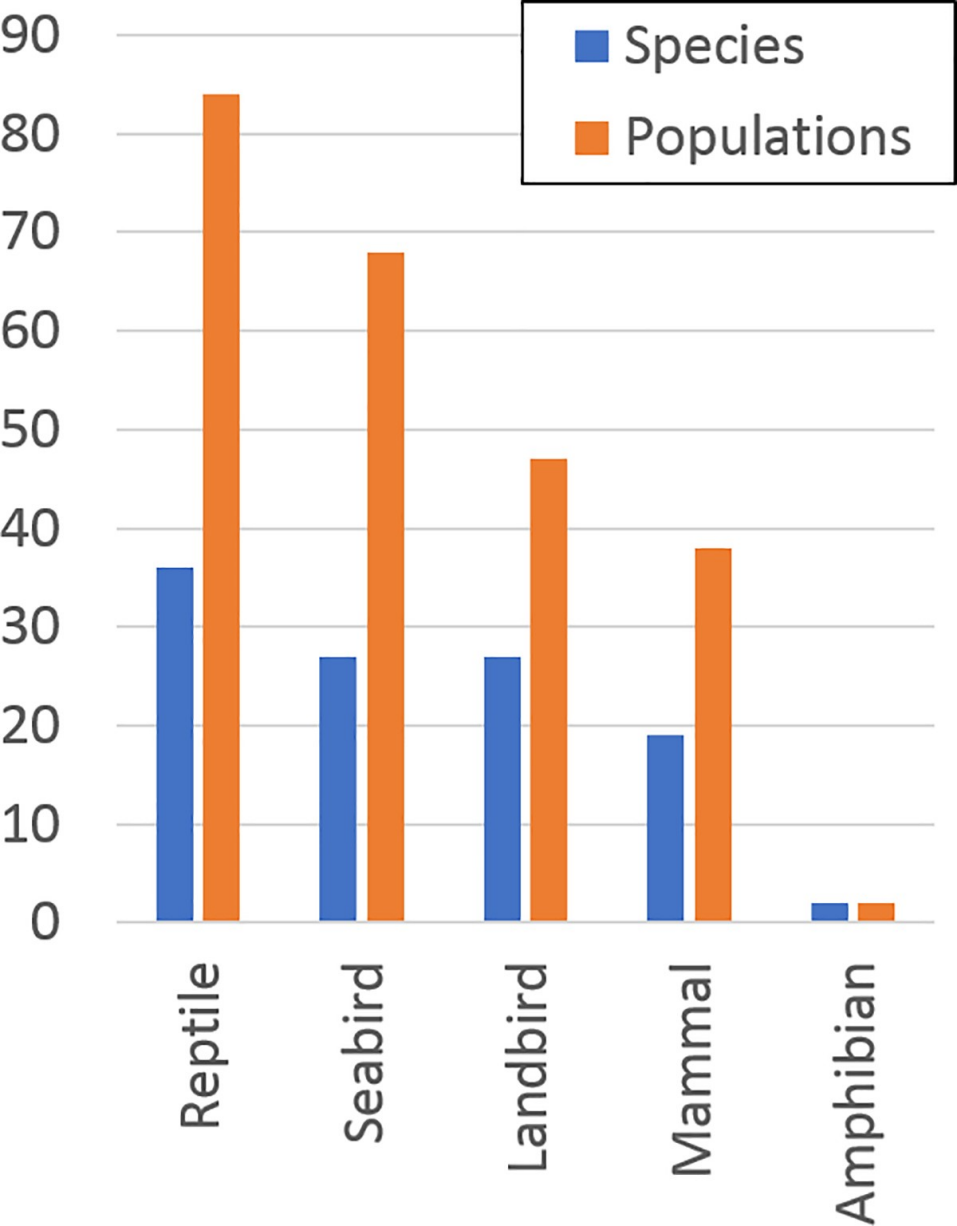
The number of highly threatened reptile, seabird, landbird, mammal and amphibian species and populations on islands where eradication of invasive mammals could feasibly be initiated by 2020 or 2030.

In total, 107 islands were identified as both technically and socio-politically feasible for initiating an eradication by 2020. These islands occurred within 34 countries and territories (S1 Data File), particularly in Mexico, French Polynesia (France), the Northern Marianas (USA), and countries and territories in the Caribbean region (Fig 1). These eradications would potentially benefit 151 populations of 80 species of highly threatened native vertebrates on islands globally. The median island size of the 107 islands was 1.11 km^2^, with a total area of 3,273 km^2^, and 69% of these islands are uninhabited by people. A median of one invasive mammal species was present on each island (maximum = 7), with *Rattus* spp. the most common taxon. Eradications on the highest-ranking eight of these islands would benefit 24 populations of 23 highly threatened species (Table 1). Another 62 islands in 25 countries and territories were considered socio-politically feasible by 2030, benefiting an additional 88 populations of 51 species of highly threatened native vertebrates on islands globally (Fig 2). Forty-nine islands in 17 countries and territories were not considered feasible in the foreseeable future, with 72 populations of 42 species that will require alternative conservation measures in the near term to prevent imminent extinctions.

**Table 1:** Details of the highest-ranked islands where eradication of invasive mammals could feasibly be initiated by 2020 and would deliver the greatest benefit to conservation of highly threatened (CR and EN) native vertebrate species. Asterisks (*) reflect invasive mammal populations currently subject to on-going eradication operations or awaiting confirmation of the outcome.

Island name	Country/ territory	Invasive mammal species having a negative impact on threatened species, and meeting eradication criteria	Threatened species benefiting from the eradication of these invasive mammals
Socorro	Mexico	Felis catus*, *Mus musculus*	*Mimus graysoni*, *Urosaurus auriculatus*, *Puffinus auricularis*
San José	Mexico	*Canis familiaris*, *Capra hircus*, *Felis catus*	*Dipodomys insularis*, *Sylvilagus mansuetus*
Gough	St Helena, Ascension and Tristan da Cunha	*Mus musculus*	*Rowettia goughensis*, *Diomedea dabbenena*, *Phoebetria fusca*, *Thalassarche chlororhynchos*, *Pterodroma incerta*
Mona	Puerto Rico	*Capra hircus*, *Felis catus*, *Rattus rattus*, *Sus scrofa*	*Agelaius xanthomus*, *Epicrates monensis*, *Cyclura stejnegeri*, *Spondylurus monae*, *Typhlops monensis*
Floreana	Ecuador	*Bos taurus*, *Canis familiaris*, *Equus caballus*, *Felis catus*, *Mus musculus*, *Rattus rattus*	*Camarhynchus pauper*, *Pterodroma phaeopygia*, *Spheniscus mendiculus*
Amsterdam	French Southern Territories	*Felis catus*, *Mus musculus*, *Rattus norvegicus*	*Diomedea amsterdamensis*, *Phoebetria fusca*, *Thalassarche carteri*, *Eudyptes moseleyi*
Alejandro Selkirk	Chile	*Bos taurus*, *Capra hircus*, *Felis catus*, *Mus musculus*, *Rattus norvegicus*, *Rattus rattus*	*Aphrastura masafuerae*
Niau	French Polynesia	*Felis catus*, *Rattus rattus*	*Todiramphus gambieri*

## Discussion

### Globally important islands for preventing extinctions

Islands offer an important opportunity for global biodiversity conservation gains because they host a larger number of threatened and endemic species per unit area than continents [23, 37]. For many island species, eradicating invasive mammals may be more achievable than other actions to reduce key threats, such as habitat loss and pollution. Our study is the first global attempt to assess opportunities for initiating eradications in the near future within both a technical and a socio-political evaluation framework. While we consider this framework appropriate for conservation planning at a global scale, it would not represent a complete feasibility assessment for an eradication operation at an island scale. To be realized, potential eradication projects that we identify will need to be assessed at a finer resolution, including consideration of regulatory, socio-political, technical, and ecological factors [24, 29, 38], allowing context-specific consideration of barriers to success, environmental impacts, and other risks to threatened species survival. Our study provides a global perspective of opportunities for highly threatened vertebrates on islands through the conservation action of invasive mammal eradication.

Eradicating invasive mammals from the 169 globally important islands that we identified as socio-politically feasible to initiate by 2020 or 2030 would improve the survival prospects of 9.4% of the Earth’s most highly threatened terrestrial insular vertebrates (111 of 1,184 species). Concentrating efforts on the 107 islands in 34 countries and territories identified as feasible to initiate by 2020 would benefit 151 populations of 80 highly threatened vertebrates and make a considerable contribution towards meeting global biodiversity targets. These results underscore the value of including invasive mammal eradication on islands within global strategies developed by (a) parties to multilateral treaties, such as the Convention on Biological Diversity (CBD) [12] for 2020 and post-2020 planning horizons, and intergovernmental organizations such as the United Nations Sustainable Development Goals [13], and (b) funders supporting biodiversity conservation, such as the Global Environment Facility [14], the World Bank and a range of private foundations. In this regard, we highlight the remaining period of the CBD 2011–2020 Biodiversity Strategy, and the value of these 107 islands for contributing to Targets 9 and 12. Likewise, including action toward invasive alien species on priority islands would bring a beneficial focus to post-2020 CBD targets.

### Supporting conservation decision-making at national scales

Our results support national conservation efforts by highlighting globally important islands where project opportunities exist to aid recovery of highly threatened vertebrates, and are thus important for National Biodiversity Strategies and Action Plans (e.g. [15]) and to support country commitments to global conservation targets [12]. These 107 islands occur in 34 countries and territories, 64% of which are classified by the World Bank as high-income economies, indicating likelihood for some financial capacity at a national scale exists to support eradication projects. Many of these islands occur in biodiversity hotspots [39], e.g. Caribbean and French Polynesia, overlapping with national strategies to reduce biodiversity loss. It is at these national scales that decisions regarding natural resources are typically made, legislation and policy contexts best apply, and local considerations can be better identified and represented. Our global analysis draws attention to the world’s most important islands for preventing imminent extinctions but should be viewed as the beginning of a longer process needed to assess eradication feasibility at local scales. For example, whereas this global analysis ascribes equal conservation value to all highly threatened vertebrate species, at local scales these species may not be equally valued, due to biological, socio-economic or political factors. Furthermore, prioritization incorporating optimization and complementarity, whereby return on investment is assessed given target objectives [40, 41], is best examined at regional or national scales.

Financial cost is another important factor that can influence feasibility [34] and has been effectively incorporated into other prioritizations for invasive vertebrate eradication on islands at regional scales, where more rigorously defensible assumptions can be made within one political and geographic area [41, 42]. Inclusion of costs in global studies like ours is less relevant because there is no single global budget that is available to be apportioned. Nevertheless, broad cost estimates can be approximated to help contextualize feasibility of projects. Almost half (n = 45) of the 107 globally important islands for invasive mammal eradication are small (<100 ha), uninhabited and require only one (n = 38) or two (n = 7) invasive mammal species to be eradicated (S1 Data File). These are factors associated with lower eradication project complexity, and therefore lower cost. Such eradications would benefit 24 highly threatened vertebrates. The list of 107 islands also includes projects with higher complexity, and therefore higher cost–including 15 larger islands (>1,000 ha), permanent human habitation and up to six invasive mammal species–but yield opportunities to benefit a different set of 24 highly threatened vertebrates. These eradications on inhabited islands could also deliver tangible benefits to the quality of life of local communities [43–45], such as substantial economic [46], ecosystem service [43] and health benefits [47], benefits to marine resources [48], and contribution toward sustainable development [13]. For example, the eradication of cats and rodents from Floreana Island, Galapagos, is identified as necessary to improve conditions for nature-based tourism, sustainable agriculture and reducing disease risk to humans [49].

Re-invasion risk and post-project biosecurity are key considerations for the feasibility of invasive mammal eradication programs on islands [36] that are primarily incorporated at local or regional scales. We did not include re-invasion risk, nor risk from new invasive mammal invasions, within our feasibility assessment because of the scarcity and inconsistency of data available for our global-scale analyses. Instead, we erred on the side of caution by including all islands with highly threatened vertebrates, regardless of reinvasion risk or biosecurity, so they can be considered for further assessment. In the assessment of sociopolitical feasibility, this contributed to some island eradications being classified as not in the foreseeable future. In other cases, where re-invasion may be expected to occur, the conservation value of these islands can be high enough to justify eradication combined with a strong biosecurity program, such as regular monitoring and prophylactic treatment (e.g. Nonsuch Island, Bermuda; or Mejía, Mexico). Ultimately, we expect that measures to evaluate and offset re-invasion risk need to be tailored to each individual island, based upon principles of biosecurity [36, 44].

Finally, eradication programs require island-specific planning in a whole-ecosystem context to determine overall feasibility [50]. Detrimental or unwanted changes to other ecosystem components can occur and should be anticipated using the best available data and logic [51]. Ultimately, eradication programs should only proceed when the expected environmental benefits exceed the non-monetary costs (social and environmental).

### Invasive mammal eradication on islands as a key conservation action

A number of important islands from our analysis are already subject to island-specific feasibility assessments [52–54], and represent globally important opportunities for the conservation of highly threatened species. Between the time of data collation and publication, several of the 107 islands have had an invasive mammal eradication successfully completed, including South Georgia (United Kingdom), Choros (Chile) and Cabritos (Dominican Republic) islands, providing practical context for our results and highlighting the importance of these nations’ efforts to protect globally threatened species. For islands we identified as not feasible in the foreseeable future, advancing the application of existing techniques on more challenging projects, or developing new techniques that overcome existing limitations, may change this circumstance [55].

Eradication of invasive mammals is often considered a baseline activity necessary to advance broader island restoration goals. Removing the threats of predation and habitat modification can provide direct benefits to threatened species; however, additional conservation and restoration actions may be necessary to fully realize recovery targets [56], such as habitat restoration, assisted colonization, conservation translocations, or control of other invasive species for which eradication techniques may not yet exist. Further, for those islands not considered feasible due to island area or human population size, sub-island conservation actions such as predator-proof fencing, or conservation activities on satellite islets (e.g. conservation translocation of highly threatened species plus eradication of invasive mammals), may help to conserve and provide new refuges for threatened species at risk from invasive mammals [57].

Impacts from climate change threaten many species and are important to consider for proposed island restoration projects [58–60]. While highly threatened vertebrates only occurring on low-lying islands are at greater risk from climate change (e.g. Polynesian ground-dove *Alopecoenas erythropterus*), the threat from invasive mammals remains significant [61] and could drive the species extinct before sea level rise and climate change-driven habitat loss become important. We suggest that removing the threat from invasive mammals in the near future can increase the resilience of highly threatened vertebrate populations on islands to projected impacts from climate change (e.g. [48]), or buy a species critical time before another conservation intervention will be necessary [62]. Eradication of invasive species is therefore an essential intervention as part of longer-term conservation strategies for these species. We expect that identifying high elevation islands where eradication of invasive mammals is feasible, or islands where appropriate habitat is expected to persist or can be created, will be a valuable consideration for long-term planning for the persistence of species that are currently restricted to low elevation islands.

Eradicating invasive mammals from these 169 islands would benefit conservation beyond the highly threatened species we identified. By removing the threat of the invasive species, efforts to reestablish extirpated species can be undertaken [9], such as for the globally threatened Raso lark (*Alauda razae*) recently re-established on Santa Luzia, where feral cat eradication is underway, and Floreana Mockingbird (*Mimus trifasciatus*) and Socorro Dove (*Zenaida graysoni*) to their namesake islands, following invasive mammal eradications. We also expect that other threatened species on restored islands will benefit, including those considered Vulnerable or Near Threatened on the IUCN Red List, plus lesser-known taxa of conservation need, including invertebrates, unlisted reptiles and plants. Addressing the conservation status and impact from invasive mammals across these species’ ranges is an important priority and would help to identify other globally important islands to undertake invasive mammal eradications. Nonetheless, evidence to date indicates that threatened bird and mammal diversity is a good indicator of threatened plant and invertebrate diversity on islands [63]. Thus, eradicating invasive mammals from the priority islands we identify would also benefit many threatened island plants and other animals, and provide globally significant conservation outcomes.

Our results identify 169 of the most globally important islands where eradication of invasive mammals by 2020 or 2030 meet criteria for technical and socio-political feasibility and can aid extinction prevention for nearly 10% of highly threatened vertebrates. A key next step for many of these islands will be fine-scale feasibility study and implementation planning at the island-scale. Where such investigations confirm the appropriateness of eradication efforts, commitment by national and local authorities and non-government partners to implement actions will be key, and we recommend integration of these projects into archipelago or region-scale programs for biodiversity conservation and sustainable development. We also recommend that the eradication of invasive species, particularly mammals on islands, and biosecurity to prevent new invasive species populations from becoming established, be explicitly identified in post-2020 biodiversity targets being negotiated by the CBD as a focused opportunity to advance global biodiversity conservation goals and prevent further extinctions.

## Supporting information

S1 Data File Table of islands, country or territory of ownership, invasive mammals and highly threatened species occurring on island, island rank reflecting conservation value, and timeframe assessed by socio-political survey in which an eradication could feasibly be initiated. Invasive mammal species listed are only those identified as having negative impact on highly threatened species and which occur on islands that fall below island area and human population size thresholds used in the analyses. Threatened species are only those that would benefit from the eradication. Stars * reflect invasive species populations currently subject to on-going eradication efforts or awaiting determination of the outcome from a completed eradication. Island names identified as unknown are deliberate to prevent revealing locations of sensitive species.
(XLSX)

S2 Data File Table of islands where no socio-political feasibility data was available during this study, country or territory of ownership, invasive mammals and highly threatened species occurring on island. Invasive mammal species listed are only those identified as having negative impact on highly threatened species and fall below island area and human population size thresholds used in the analyses. Threatened species are only those that would benefit from the eradication. Island names identified as unknown are deliberate to prevent revealing locations of sensitive species.
(XLSX)

S1 File Additional figures, tables and text supporting the main paper. (DOCX)
