# Supplementary material for: Globally important islands where eradicating invasive mammals will benefit highly threatened vertebrates
Source: PLoS One. 2019 Mar 27;14(3):e0212128. doi: 10.1371/journal.pone.0212128 (PMC6436766; doi:10.1371/journal.pone.0212128)
Supplement: S1 File — (DOCX) [file pone.0212128.s003.docx]

**SUPPORTING INFORMATION**

**Globally important islands for eradicating invasive mammals to benefit highly threatened vertebrates**

Nick D. Holmes^*^, Dena R. Spatz, Steffen Oppel, Bernie Tershy, Donald A. Croll, Brad Keitt, Piero Genovesi, Ian J. Burfield, David J. Will, Alexander L. Bond, Alex Wegmann, Alfonso Aguirre-Muñoz, André F. Raine, Charles R. Knapp, Chung-Hang Hung, David Wingate, Erin Hagen, Federico Méndez-Sánchez, Gerard Rocamora, Hsiao-Wei Yuan, Jakob Fric, James Millett, James Russell, Jill Liske-Clark, Eric Vidal, Hervé Jourdan, Karl Campbell, Keith Springer, Kirsty Swinnerton, Lolita Gibbons-Decherong, Olivier Langrand, M. de L. Brooke, Miguel McMinn, Nancy Bunbury, Nuno Oliveira, Paolo Sposimo, Pedro Geraldes, Pete McClelland, Peter Hodum, Peter G. Ryan, Rafael Borroto-Páez, Ray Pierce, Richard Griffiths, Robert N. Fisher, Ross Wanless, Stesha A. Pasachnik, Steve Cranwell, Thierry Micol, Stuart H. M. Butchart

* Corresponding author

E-mail: [nick.holmes@islandconservation.org](mailto:nick.holmes@islandconservation.org) (NDH)

**Table A:** Impact classification of invasive species on highly-threatened vertebrates.

| **Invasive species impact** | **Definition** |
| --- | --- |
| Confirmed | Documented in BirdLife International (2013) as causing a high-, medium- or low-impact threat on the specified native bird species; OR evidence from experts and/or literature confirms that the invasive species impacts the highly-threatened vertebrates (at any threatened vertebrate breeding location). |
| Suspected | An impact from the invasive species to the highly-threatened vertebrate is suspected based on the literature or by experts, but it has never been documented. |
| None | No evidence of impact from invasive species to the highly-threatened vertebrate or confirmed that no impact occurs based on sources above. |

**Table B**: Area and / or human population size thresholds for feasibility of eradications used in analyses, based on data from [1], using successful and planned / underway whole island eradications with data quality classified as good or satisfactory. We used successful eradications to date as thresholds and supplemented these with area and human population size from known eradications planned or underway when they exceeded current values. See text in S1 File for methods and the sensitivity analyses comparing results using these thresholds. Human population size based on ordinal categories. *Excludes American Mink, and the on-going effort to eradicate Small Indian Mongoose from Amami-Ōshima.

| **Invasive animal category** | **Island area (ha) (successful)** | **Human population size (successful)** | **Island area (ha) (planned or underway)** | **Human population size**  **(planned or underway)** |
| --- | --- | --- | --- | --- |
| Cats | 30,000 | 1,000 | 65,000 |  |
| Civets and genets | 0 | 0 | 1,400 | 100 |
| Dogs | 90,000 | 1,000 | 90,000 |  |
| Rabbits and hares | 15,000 | 100 |  | 1,000 |
| Weasels* and mongoose | 4,000 | 100 |  |  |
| Possums | 4,000 | 100 |  |  |
| Raccoons *Procyonidae* | 100 | 0 | 5,000 | 1,000 |
| Rodent *Mus* | 15,000 | 1,000 | 30,000 |  |
| Rodent *Rattus* | 15,000 | 1,000 | 30,000 |  |
| Ungulates | 60,000 | 10,000 | 65,000 |  |

**Table C**: Spearman’s rank order correlations of historical capacity for undertaking invasive species eradications, political acceptability at a national scale, dominant use of the island, social acceptability at a local scale and timeframe to initiate an eradication. Values in italics are significant (P <0.05).

|  | **Historical Capacity** | **Political acceptability** | **Dominant Use** | **Social Acceptability** |
| --- | --- | --- | --- | --- |
| **Political acceptability** | *0.48* |  |  |  |
| **Dominant Use** | *-0.25* | *-0.26* |  |  |
| **Social Acceptability** | *0.25* | *0.71* | *-0.40* |  |
| **Potential Timeframe** | -0.11 | *-0.30* | *0.37* | *-0.44* |

**S1 Data File**: Table of islands, country or territory of ownership, invasive mammals and highly threatened species occurring on island, island rank reflecting conservation value, and timeframe assessed by socio-political survey in which an eradication could feasibly be initiated. Invasive mammal species listed are only those identified as having negative impact on highly threatened species, and which occur on islands that fall below island area and human population size thresholds used in the analyses. Threatened species are only those that would benefit from the eradication. Stars * reflect invasive species populations currently subject to on-going eradication efforts or awaiting determination of the outcome from a completed eradication. Island names identified as unknown are deliberate to prevent revealing locations of sensitive species.

(See excel sheet)

S2 Data File: Table of islands where no socio-political feasibility data was available during this study, country or territory of ownership, invasive mammals and highly threatened species occurring on island. Invasive mammal species listed are only those identified as having negative impact on highly threatened species and fall below island area and human population size thresholds used in the analyses. Threatened species are only those that would benefit from the eradication. Island names identified as unknown are deliberate to prevent revealing locations of sensitive species.

(See excel sheet)

**Fig A:** Framework used to identify globally important islands for invasive mammal eradication to benefit highly threatened vertebrates.

**
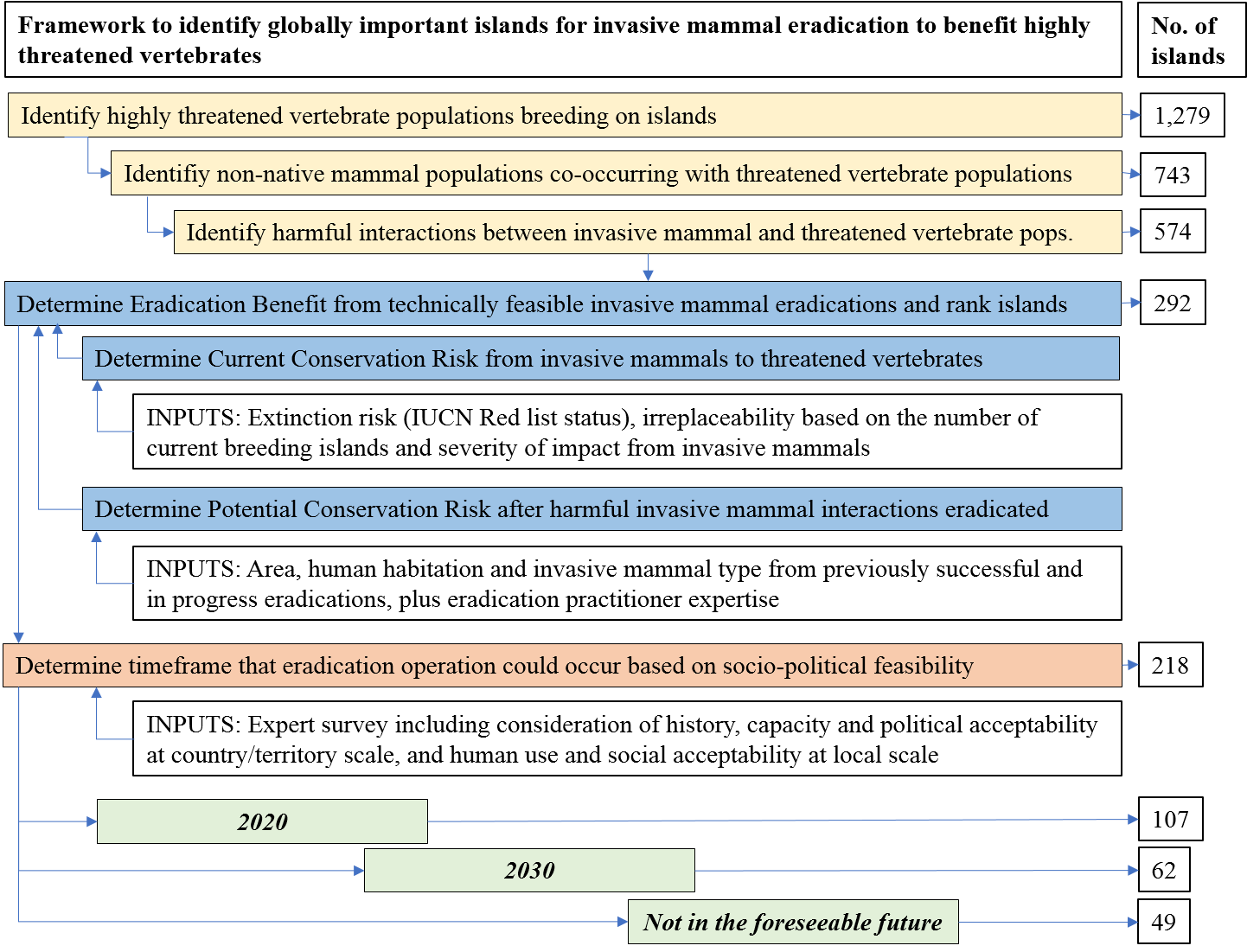
**

**Fig B:** The eight invasive mammal species with the highest number of harmful interactions. Confirmed and suspected negative interactions are in dark and light colors respectively.

**
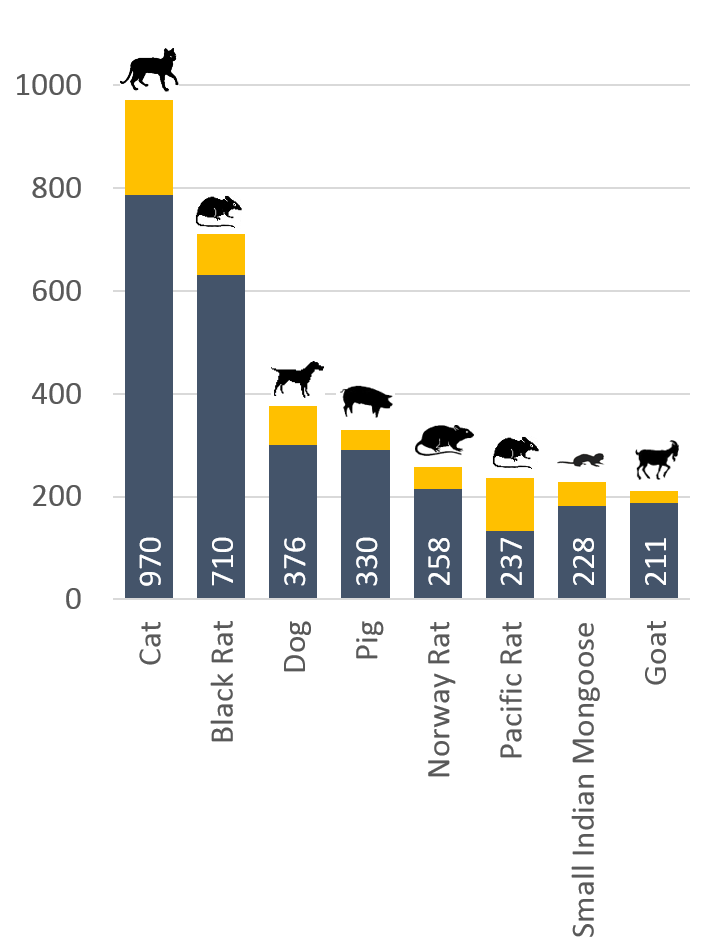
**

**Fig C:** The frequency of island ownership by timeframe within which eradications could be initiated.


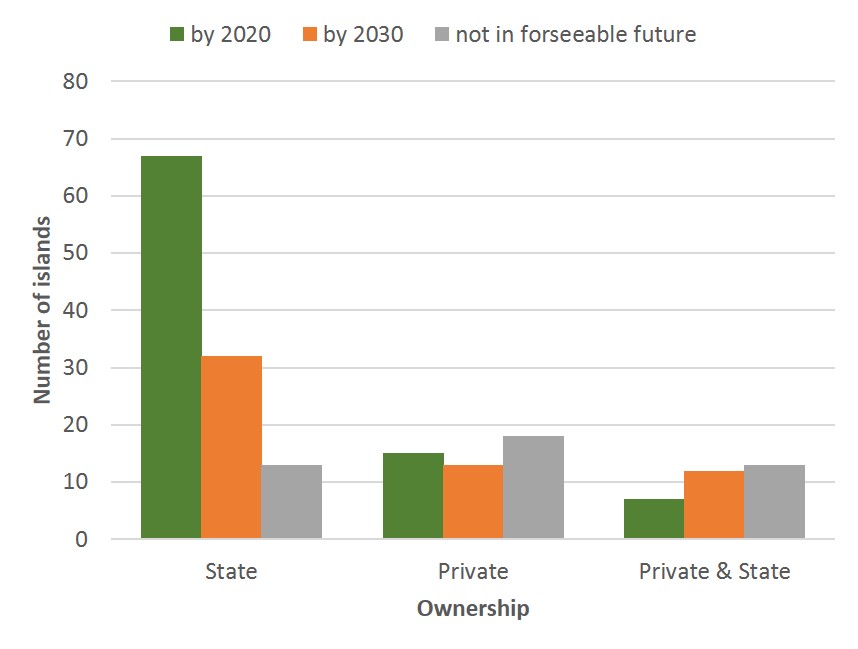


**S1 Text**

**Assessing the effect of including highly threatened vertebrates with both continental and insular populations.**

To assess the impact on our results of including these 93 species that had both insular and continental populations, we reran the analyses with fully insular species only. This resulted in 18 islands being removed from the dataset, six of which were identified as feasible to initiate by 2020 and housing a breeding population of seven highly threatened species which also have continental breeding locations (*Sterna bernsteini, Podarcis carbonelli, Dasyurus hallucatus, Pseudantechinus mimulus, Spheniscus demersus, Phalacrocorax capensis, Phalacrocorax neglectus*).

**Assessing the effect of assuming invasive rodents are present when status is unknown.**

Rodents are among the most widespread invasive mammal, and their presence can be challenging to determine. We reran the analyses and assumed that rodents were present on all islands where rodent presence was classified as unknown. This worst-case-scenario identified 142 additional islands, including 7 with a rank within the top 100 islands, where if rodents are indeed present, they are expected to be having a negative impact on populations of highly threatened vertebrates, and which fall within our basic thresholds for feasibility. This outcome highlights the need for additional surveys to determine the presence of more cryptic invasive mammals like rodents on islands with highly threatened vertebrates.

**Establishing thresholds for invasive mammal eradications.**

The data used to establish thresholds for eradication excluded events for only domestic animals, and included only whole island eradications (therefore excluding restricted range events) where data quality was scored as good or satisfactory [1]. We combined thresholds for weasels and mongoose (Herpestid or Mustelid) because techniques to eradicate these species are similar: however, we excluded American Mink (*Neovison vison*) because this species did not overlap with any highly threatened vertebrate population in our analyses, and because mink eradications have occurred on much larger islands than other mongoose or weasel eradications. Likewise, we excluded the on-going effort to eradicate Small Indian Mongoose on the Japanese Island of Amami-Ōshima from our analyses, as we considered this a unique effort given the large area (~712km^2^) and human population size (~73,000). Island area thresholds for each invasive mammal type reflect a rounded precision of 10, 100, 1000 or 5000 ha for categories of <100, 101-1,000, 1,001-10,000, or >10,000 ha, respectively. For example, an island area of 140 ha was rounded to 200 ha and 12,800 ha was rounded to 15,000 ha. For South Georgia Island we used the largest eradication unit within this operation (30,000 ha) instead of total island size given that rodents cannot move between these operational units due to glacial barriers. For Grande Terre on Aldabra Atoll we used the total atoll area given rodents are expected to move between motus on this atoll. We expressed human population size as 0,10,100, 1,000 or >10,000 based on the upper limit of the following ordinal categories of 0, 1-10, 11-100, 101-1,000, or 1,001-10,000 individuals. We subsequently sent these values to eradication experts for confirmation, in consideration of the scale and goal of the analyses. See Table B in S1 File for results.

To determine the effect of using different threshold values, we ran the analyses for thresholds based on successful eradications to date and for eradications currently in progress or planned. We excluded events classified as successful but reinvaded as these may represent misclassified operational failures. We included projects in progress or being planned because island eradication projects are continuing to increase in scope and scale, and using thresholds based only on previously successful projects would unnecessarily restrict future opportunities in the timescales we considered. This reflects a preference for a Type II error, whereby it is more valuable to retain potentially unfeasible eradications at this stage of conservation planning and discount them when more detailed island specific analyses are done, rather than to remove potentially feasible eradications from this early stage of consideration (Type I error). When using thresholds for only successful projects to date, this identified 281 islands. When using projects in progress or planned, this added 11 new islands to this list, five of which were classified as feasible by 2020, and two by 2030, in the subsequent socio-political assessment.

**Socio-political surveys and data treatment.**

Each expert received a questionnaire, which including the following questions to be considered for each island evaluated:

1. *The history of previous invasive vertebrate eradications on islands and capacity for invasive vertebrate eradications in the country or territory*. Considering whether invasive vertebrate eradications have ever been attempted in the country/territory previously, and how much experience and/or capacity exists for eradications today. Capacity includes the ability of people, organizations, and/or governments to plan, permit, finance and implement eradications. Countries or territories that have implemented no or few previous eradications are likely to need more time before a new eradication effort could be initiated. Countries or territories with more experience, and where eradication of invasive vertebrates is considered a more mainstream conservation tool, are likely to require less time before it is feasible to initiate a new program. Some capacity may exist in the absence of a history of eradications. (A list of eradications that we are aware of from the country or territory were provided based on [1]. We asked experts to identify if there was no, some or adequate capacity and coded these responses as 1,2 and 3 respectively.)
2. *Political acceptability at a national or territory level*. Considering how much experience regulators and natural resource agencies have with invasive vertebrate eradications, the current political environment, strength of environmental legislation for eradication projects, compatibility of national policies to use lethal methods for conservation outcomes, and level of bureaucracy. We expected that low political acceptability would mean that projects would take longer to overcome these barriers and could not be initiated until 2030 or perhaps not at all in the foreseeable future. (We asked experts to identify if there was low, medium or high acceptability and coded these responses as 1,2 and 3 respectively.)
3. *Influence of permanent human habitation on any proposed Invasive Alien Species (IAS) eradication*. Invasive vertebrate eradications on islands with permanent human habitation are expected to be more complex to implement and require more planning and development time before implementation. Implementation of eradications requires consent to remove animals from the entire island, so where one or multiple private individuals own land on an island this will increase complexity to meet this requirement. Typically, where human habitation includes research stations or military installations, obtaining permission is less complex because consent is typically required from management agencies only. [We asked experts to identify if there was dominant use as a) no human use, b) seasonal use or year- round military or research use, or c) year-round residents, and coded these responses as 0,1 and 2 respectively. We also provided an ‘other’ category and left this code as null. We also asked experts to classify ownership as a) government/state, b) private c) government/state and private, or d) other (neither government/state or private).]
4. *Social acceptability on a local scale*. Considering how likely is it that any human populations on the island would be engaged in an invasive species eradication project, whether there would be benefit to the primary industries on the island (e.g. tourism or agriculture), whether there is likely to be potential opposition to the use of eradication methods by island inhabitants or nearby stakeholders, and the species to be eradicated (e.g. cats are more popular than rats, deer and pigs are often hunted). We expect that low social acceptability would require greater consultation and engagement with communities and stakeholders, and mean that projects may not be possible to initiate until 2030 or perhaps not at all in the foreseeable future. (Where this is unknown, we asked experts to conservatively estimate one of the three timeframes, or identify the island as “Unknown”. We asked experts to identify if there was low, medium or high social acceptability and coded these responses as 1,2 and 3 respectively.)

We also collected ordinal data for these four factors of socio-political feasibility, for which relationships were explored using a Pearson’s rank order correlation matrix. For the nominal data of dominant use and ownership, we removed islands where we received multiple responses that conflicted (as opposed to timeframe, for which we sought consensus), and we used only summary statistics. Higher political acceptability at a national or state scale, and social acceptability at a local scale, were associated with earlier timeframes (Table C in S1 File), and for those islands with ownership data (n=190) most (76%) assessed as feasible to initiate eradication by 2020 were state-owned (Fig C in S1 File).

**References**

1. DIISE. The Database of Island Invasive Species Eradications, developed by Island Conservation, Coastal Conservation Action Laboratory UCSC, IUCN SSC Invasive Species Specialist Group, University of Auckland and Landcare Research New Zealand. <http://diise.islandconservation.org> 2014.
